# GAPTrap: A Simple Expression System for Pluripotent Stem Cells and Their Derivatives

**DOI:** 10.1016/j.stemcr.2016.07.015

**Published:** 2016-09-01

**Authors:** Tim Kao, Tanya Labonne, Jonathan C. Niclis, Ritu Chaurasia, Zerina Lokmic, Elizabeth Qian, Freya F. Bruveris, Sara E. Howden, Ali Motazedian, Jacqueline V. Schiesser, Magdaline Costa, Koula Sourris, Elizabeth Ng, David Anderson, Antonietta Giudice, Peter Farlie, Michael Cheung, Shireen R. Lamande, Anthony J. Penington, Clare L. Parish, Lachlan H. Thomson, Arash Rafii, David A. Elliott, Andrew G. Elefanty, Edouard G. Stanley

**Affiliations:** 1 Murdoch Childrens Research Institute, Flemington Road, Parkville, VIC 3052, Australia; 2 Department of Paediatrics, University of Melbourne, Parkville, VIC 3050, Australia; 3 Department of Anatomy and Developmental Biology, Monash University, Clayton, VIC 3800, Australia; 4 The Florey Institute of Neuroscience and Mental Health, Melbourne University, Parkville, VIC 3052, Australia; 5 Stem Cell and Microenvironment Laboratory, Weill Cornell Medical College in Qatar, Qatar Foundation, Education City, Doha, Qatar; 6 Department of Genetic Medicine, Weill Cornell Medical College, New York, NY 10065-4896, USA; 7 Department of Cardiology, The Royal Children's Hospital, Parkville, VIC 3052, Australia; 8 Division of Developmental Biology, Cincinnati Children's Hospital Medical Centre, Cincinnati, OH 45229, USA; 9 Australian Centre for Blood Diseases, Monash University, The Alfred Centre, Melbourne, VIC 3004, Australia; 10 School of Biosciences, University of Melbourne, Parkville, VIC 3050, Australia

**Keywords:** human pluripotent stem cells, GAPDH, reporter genes, expression system, lineage tracing, differentiation

## Abstract

The ability to reliably express fluorescent reporters or other genes of interest is important for using human pluripotent stem cells (hPSCs) as a platform for investigating cell fates and gene function. We describe a simple expression system, designated GAPTrap (GT), in which reporter genes, including *GFP*, *mCherry*, *mTagBFP2*, *luc2*, *Gluc*, and *lacZ* are inserted into the *GAPDH* locus in hPSCs. Independent clones harboring variations of the GT vectors expressed remarkably consistent levels of the reporter gene. Differentiation experiments showed that reporter expression was reliably maintained in hematopoietic cells, cardiac mesoderm, definitive endoderm, and ventral midbrain dopaminergic neurons. Similarly, analysis of teratomas derived from GT-*lacZ* hPSCs showed that β-galactosidase expression was maintained in a spectrum of cell types representing derivatives of the three germ layers. Thus, the GAPTrap vectors represent a robust and straightforward tagging system that enables indelible labeling of PSCs and their differentiated derivatives.

## Highlights

• GAPTrap vector system targets transgenes to the ubiquitously expressed GAPDH locus
• Targeting transgenes to the GAPDH locus yields reliable transgene expression
• Transgenes at this locus are robustly expressed in differentiated cells
• Generation of GAPTrap targeted human PSC lines is simple and efficient

## Graphical Abstract

**Figure:**
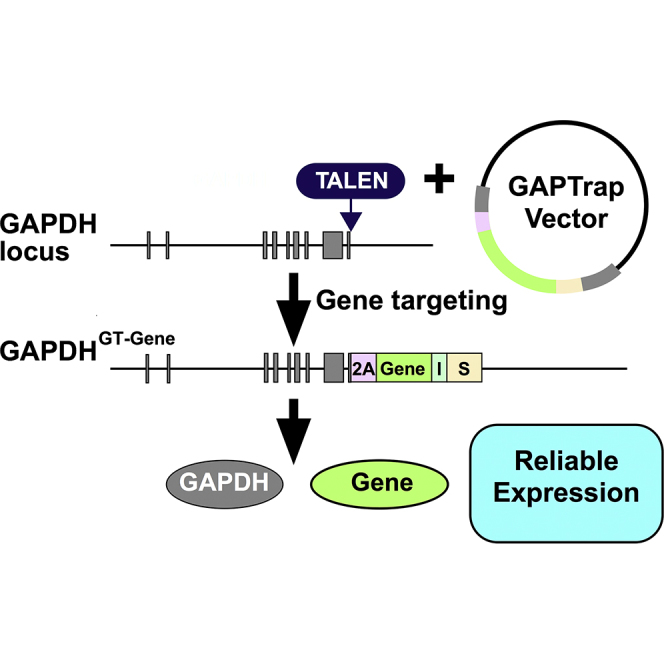

## Teaser

In this article Stanley, Elefanty, and colleagues describe a simple vector system, GAPTrap, for constitutively expressing transgenes in pluripotent stem cells and their differentiated derivatives. GAPTrap vectors target transgenes to the GAPDH locus, ensuring robust and reliable expression in a wide variety of cell types, and providing an ideal base for cell tagging and gene overexpression experiments.

## Introduction

Constitutive expression of genes in pluripotent stem cells (PSCs) and their differentiated progeny has been a critical tool for studying gene function both in vivo and in vitro. In the mouse, the most widely used system targets *Rosa26*, a locus identified in an embryonic stem cell (ESC) gene-trapping experiment (Zambrowicz et al., 1997). The original Rosa26-lacZ reporter gene displayed ubiquitous and reliable expression in all tissues of ESC-derived mice, prompting researchers to subsequently modify this locus to enable expression of other genes and genetic elements (Soriano, 1999). Although ubiquitously expressed loci in human PSCs (hPSCs) have been described (Costa et al., 2005, Irion et al., 2007), few studies have utilized these for overexpression or lineage-tracing studies. More recently, a number of laboratories have reported successful introduction of gene-expression cassettes into the Adeno-Associated Virus 1 (AAVS1) integration site locus (Luo et al., 2014, Qian et al., 2014, Smith et al., 2008). In these studies, transgenes in this locus maintain reliable expression in a number of differentiated lineages and, in some instances, expression can be regulated through inclusion of tetracycline-responsive elements (Hockemeyer et al., 2009, Sim et al., 2015). The demonstrated utility of the AAVS1 system provides impetus for the development of alternative expression platforms, providing researchers with an opportunity to undertake increasingly sophisticated experiments requiring multiple genetic modifications.

The glyceraldehyde 3-phosphate dehydrogenase (*GAPDH*) gene encodes a key component of the glycolytic pathway, a process that turns glucose into ATP in all cells. GAPDH is ubiquitously expressed at consistently high levels in many different cell types, making it an ideal gene upon which to base a gene-expression system (Barber et al., 2005) (GTex Consortium, 2015, Murphy and Polak, 2002) (http://www.gtexportal.org/home/gene/GAPDH). We have generated a series of vectors in which the expression of introduced genes is derived from the *GAPDH* transcript, ensuring high-level, widespread expression of the transgene. In addition, our vector design links expression of a selectable marker gene to integration dependent trapping the *GAPDH* promoter, greatly enhancing the probability of obtaining correctly targeted clones.

## Results

**Figure 1:**
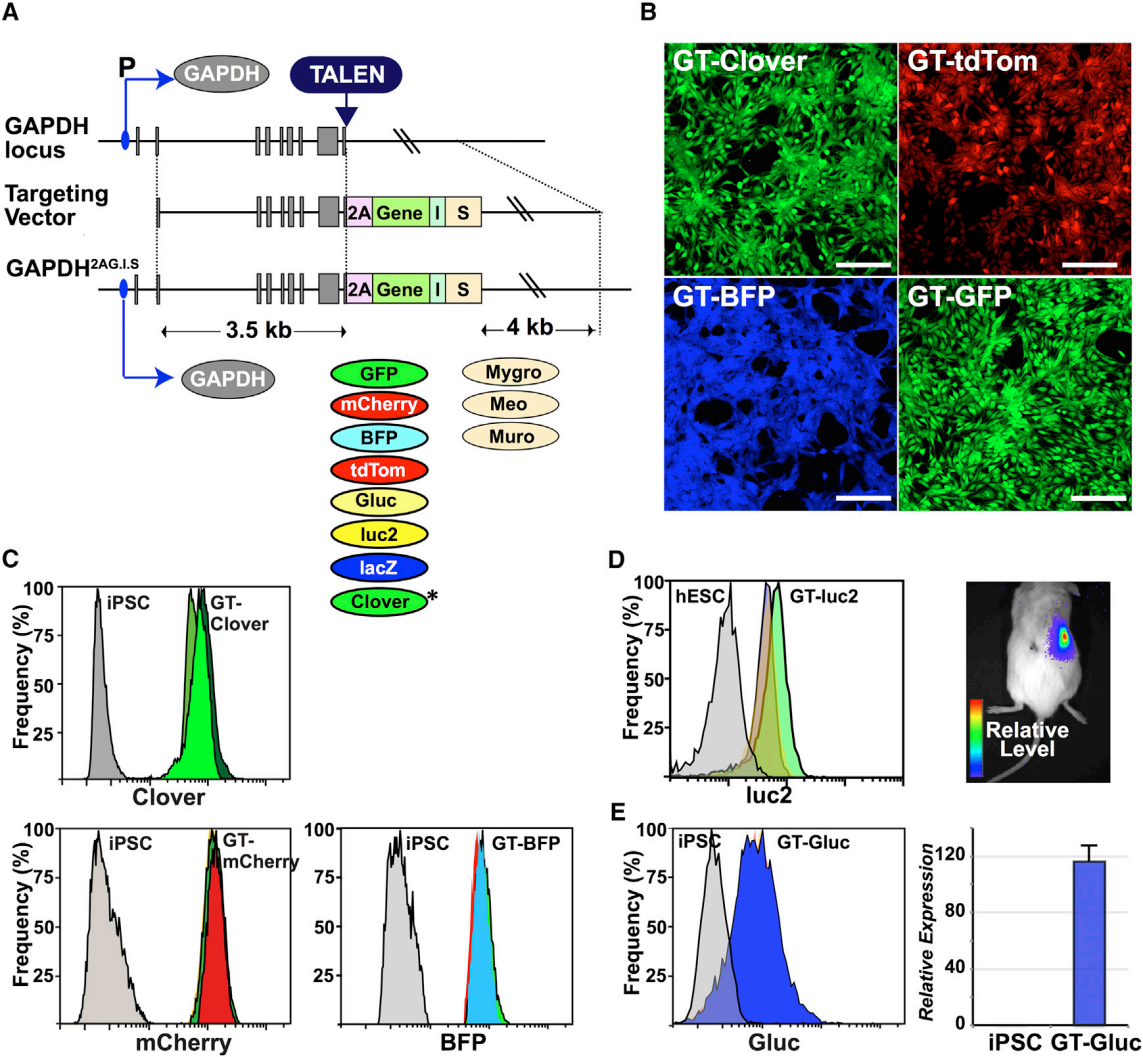
Structure and Function of the GAPTrap Vectors and Selected Variants (A) GAPTrap (GT) vector structure relative to the *GAPDH* locus. The upper line represents the *GAPDH* locus with exons shown as gray boxes, with the promoter (P) and position of the TALEN target sites marked. The middle line shows the GT targeting vector, comprising from 5′ to 3′: a 5′ *GAPDH* homology arm, a T2A peptide sequence (2A) fused in frame with the GAPDH coding sequence, the gene of interest (Gene), an internal ribosomal entry site (I), a selectable marker (S), and a 3′ *GAPDH* homology arm. The lower line shows the structure of the modified *GAPDH* locus, with the genes tested in this study shown below. These are EGFP (GFP), mCherry, mtagBFP2 (BFP), Tandem tomato (TdTom), *Gaussia* luciferase (GLuc), firefly luciferase (Luc2), β-galactosidase (LacZ), and Clover. The three selectable markers (S) encoding hygromycin (Mygro), G418 (Meo), and puromycin (Muro) are also shown. Asterisk denotes that in the GT-Clover vector the selectable marker is positioned after a second T2A sequence. (B) Confocal microscopy fluorescence images of undifferentiated hPSCs expressing Clover, Tandem tomato, mtagBFP2, and GFP. Scale bars, 200 μm. (C) Flow cytometry histograms showing clone-to-clone consistency of expression for three independent hPSC lines containing GT-Clover, GT-mCherry, GT-BFP vectors. (D) Intracellular flow cytometry of three independent hPSC lines containing the GT-Luc2 expression cassette (left) and the results of bioluminescent imaging showing a localized signal from GT-Luc2 hPSCs transplanted subcutaneously in an immunodeficient mouse (8 days post transplant) (right). (E) Flow cytometry histograms showing clone-to-clone consistency for three independent hPSC lines expressing *Gaussia* luciferase (GLuc) (left) and a summary of results of luciferase assays for the three GT-GLuc clones. The graph shows the mean relative expression and the SEM. Luciferase activity is absent from the unmodified parental iPSC.

The structure of this GAPTrap (GT) vector is shown in Figure 1A. Genes of interest are inserted in frame with a T2A sequence (Szymczak and Vignali, 2005, Szymczak et al., 2004) that replaces the *GAPDH* stop codon. An internal ribosome entry site (Jang et al., 1990) located immediately downstream is used to express selectable marker genes encoding neomycin, hygromycin, or puromycin resistance. In the GT vectors, sequences encoding these antibiotic resistance markers have been optimized for expression in mammalian cells and are designated *Meo*, *Mygro*, and *Muro*. Vectors, Addgene accession numbers and cell lines generated for this study are listed in Table S1.

To assist with the generation of targeted clones, we utilized transcription activator-like effector nucleases (TALENs) (Wood et al., 2011, Hockemeyer et al., 2011) or the CRISPR/Cas9 system directed against the point within the *GAPDH* locus at which the vector is inserted. We found that introduction of a double-stranded break using either TALENs or CRISPRs was essential for the generation of correctly targeted clones. Depending on the vector configuration, targeting efficiencies were often greater than 70%. For example, GT-TdTom and GT-lacZ vectors gave targeting frequencies of 10/12 (83%) and 9/10 (90%) when using TALENs (see Figure S1C). Similarly, CRISPR/Cas9-assisted homologous recombination yielded a targeting efficiency of 80% (12/15) (Figure S1D). To ascertain the frequency at which insertions and deletions (Indels) occurred in the unmodified GAPDH allele of cells containing a GT vector, we sequenced the region corresponding to the point within the GAPDH allele targeted by the GAPDH TALENs. This analysis showed that of 24 GT-reporter lines, 25% had Indels, indicative of non-homologous end-joining (NHEJ) events. Given the relatively low frequency of on-target NHEJ events in the GAPDH locus itself, the presence of off-target cutting events by this pair of TALENs was not assessed.

GAPDH functions as a tetramer, and examination of the 3D crystal structure indicated that the C terminus of each GAPDH subunit is located on the exterior surface of the tetramer (Ismail and Park, 2005), suggesting that additional amino acids encoded by the T2A sequence should not interfere with enzymatic activity. However, examination of GAPDH protein using western blot analysis indicated that modified alleles that include an internal ribosome entry site (IRES)-selectable marker cassette were expressed at lower levels than the wild-type GAPDH allele. Thus, although we expect the specific activity of GAPDH-T2A proteins to be the same as that of the wild-type protein, the reduced expression of GAPDH-T2A from the modified allele may explain why cells with two targeted GAPDH alleles could not be isolated (Figure S1E).

Using the vectors shown in Figure 1A, we generated a series of PSC lines that expressed EGFP (Cormack et al., 1996), Clover (Lam et al., 2012), mCherry (Merzlyak et al., 2007), mtagBFP2 (Subach et al., 2011), Tandem tomato (tdTomato) (Shaner et al., 2004), luciferase 2 (Luc2) (Promega), a membrane-bound *Gaussia princeps* luciferase (GLuc) (Santos et al., 2009), and nuclear LacZ (Stanley et al., 2000) (Table S1). Cells expressing the fluorescent markers could be readily visualized by microscopy (Figure 1B).

Flow cytometry analysis showed that independent targeted clones expressed remarkably similar levels of the inserted reporter genes, highlighting the consistency of expression produced by this vector configuration (Figure 1C). This consistency also enabled us to conclude that all the selectable marker genes adversely affected expression of the upstream fluorescent reporter (Figure S1F). Intracellular flow cytometry of GT-Luc2 H9 cells showed that independent clones also expressed *Luc2* at consistent levels while transplantation experiments indicated expression was sufficient to observe subcutaneously grafted cells using bioluminescent imaging (Figure 1D). Similarly, surface expression of GLuc could be detected on GT-GLuc iPSCs using flow cytometry, and consistent levels of GLuc activity could be readily assayed on live cells (Figure 1E).

**Figure 2:**
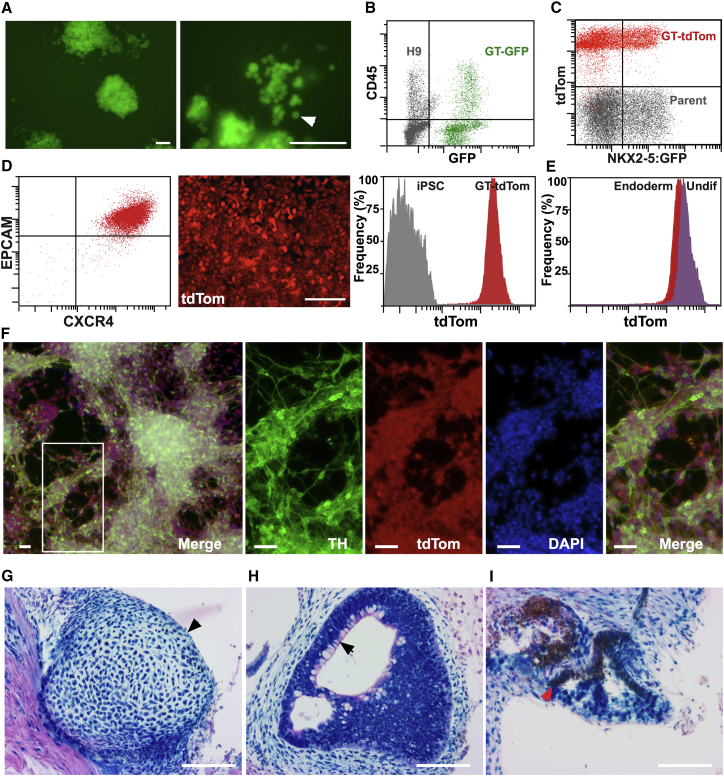
Maintenance of Reporter Expression during Differentiation of GAPTrap hPSCs (A) Fluorescence images of GFP-expressing day-14 hematopoietic colonies formed in methylcellulose. The right panel shows that individual blood cells can be readily identified (white arrow). Scale bars, 100 μm. (B) Flow cytometry analysis showing that all cells isolated from the cultures in (A) maintain GFP expression. (C) Flow cytometry analysis of differentiation day 10 NKX2-5^+^ cardiomyocytes derived from parental and GT-TdTom tagged hESCs. (D) Differentiation of GT-TdTom iPSCs to CXCR4^+^EpCAM^+^ endoderm shows that Tandem tomato (tdTom) expression is clearly detectable by fluorescence microscopy and is maintained at uniform levels, as determined by flow cytometry. Scale bars, 200 μm. (E) Flow cytometry histograms showing that the level of Tandem tomato expression is similar in undifferentiated (Undiff) GT-TdTom iPSCs and their endodermal derivatives. (F) Fluorescence microscopy images showing expression of GT-TdTom in tyrosine hydroxylase (TH)-positive neurons at differentiation day 30. The TdTom panel shows Tandem tomato fluorescence following fixation and processing for antibody staining with anti-TH antibodies. DAPI was used to visualize nuclei. Scale bars, 100 μm. (G–I) Bright-field images of eosin-counterstained sections from teratomas derived from GT-lacZ iPSCs that were stained with X-gal to reveal lacZ activity prior to sectioning. Arrowheads indicate cartilage (mesoderm) (G), goblet cells (endoderm) (H), and pigmented epithelium (ectoderm) (I). Scale bars, 100 μm.

We examined the reliability of expression during the differentiation to derivatives of the three embryonic germ layers, mesoderm, endoderm, and ectoderm. These studies indicated that robust expression was maintained when GT-GFP H9 hESCs or GT-tdTomato iPSCs were differentiated to hematopoietic mesoderm, with readily detectable expression in individual hematopoietic cells by fluorescence microscopy and flow cytometry (Figures 2A, 2B, and S2A). Similarly, tdTomato expression from the *GAPDH* locus (GT-TdTom) was maintained at robust levels in cardiac mesoderm at differentiation day 7, identified by expression of GFP from the *NKX2-5* locus (Elliott et al., 2011) (Figure 2C). Bright GT-tdTomato expression was also observed in CXCR4^+^EpCAM^+^ definitive endoderm cells at differentiation day 4 (Figures 2D and S2B), with expression levels similar to that seen in undifferentiated GT-tdTomato PSCs (Figure 2E). Using a neural differentiation protocol, we observed strong and consistent tdTomato expression in tyrosine hydroxylase (TH)-positive ventral midbrain dopamine neurons at differentiation day 30 (Figure 2F, see also GT-GFP expression in Figure S2C).

To further explore the spectrum of cell types that expressed the GT-reporter system, we generated teratomas from GT-lacZ PSCs by subcutaneous injection of 10^6^ undifferentiated cells into NOD/SCID/IL2Rγ null mice (Shultz et al., 2005). After 2–3 months, palpable teratoma masses were removed, fixed in paraformaldehyde, and subsequently stained with X-gal to reveal cells expressing β-galactosidase activity. This analysis revealed that the surface of teratomas and the surface of dissected teratoma subfragments stained strongly and uniformly with X-gal (Figure S2D). Examination of eosin-stained sections revealed a variety of differentiated tissue types, including cartilage (Figure 2G), epithelium containing goblet-like cells and surrounding mesenchyme (Figure 2H), and pigmented epithelium (Figure 2I) that displayed strong X-gal staining, suggesting the GT-lacZ transgene continued to be expressed, even after an extended period of differentiation in vivo.

## Discussion

We have generated a series of vectors designated GAPTrap, or GT, based on the *GAPDH* locus, a ubiquitously and robustly expressed housekeeping gene. We have constructed and tested a range of reporters and have shown that their expression is maintained in a wide variety of cell types, and for longer than 2 months in vivo, making the system ideal for the long-term tracking of cells in vitro and subsequent transplantation. In addition to commonly used fluorescent proteins, we have tested a number of other reporters we believe will be useful for in vivo cell tracking. For example, luciferase (Luc2) allows the visualization of cells in living animals using bioluminescent imaging. As stem cell technologies transit the preclinical testing phase of development, such imaging systems will be useful for understanding the behavior of differentiated cells in animal models. We also found that a previously described surface-directed luciferase, GLuc (Santos et al., 2009), was robustly expressed, despite the fact that the signal sequence required for trafficking to the cell membrane was C-terminal of cytoplasmic GAPDH. Lastly, we have illustrated how a GT-lacZ expression system enables the simple detection of PSC-derived cells in a context compatible with standard paraffin-embedded histological sections.

In addition to configurations for constitutive gene expression, we also tested the behavior of the Tet-on system (http://www.clontech.com) in which sequences encoding either GFP or mCherry expression were placed under the control of a doxycycline-responsive promoter. Although robust doxycycline-inducible expression was observed in early passage lines, the vectors eventually underwent silencing. It is not clear whether this silencing was an inherent property of the genomic position of the transgene or a function of the vector configuration. Whatever the underlying mechanism, silencing or variegated expression of transgenes that contain either inducible or tissue-specific promoters seems to be an issue affecting other “safe harbor” loci (Ordovas et al., 2015).

Taking into account the 3D structure of GAPDH, we hypothesized that the presence of 2A peptide at the C terminus would not interfere with GAPDH function. However, western blot analysis indicated a clear reduction in the level of GAPDH-T2A protein relative to GAPDH protein translated from the unmodified *GAPDH* allele. Analysis of different vector configurations suggested that this lower level of expression was in part due to sequences downstream of the reporter gene. We observed that expression of GFP from different variants of the GT vectors differed substantially, depending on the sequence of the selectable marker 3′ of the IRES. Indeed, in vectors that lacked the IRES-selectable marker cassette altogether, the intensity of GFP was almost ten times brighter than in cells containing a GT vector that included the IRES-Mygro cassette (Figure S1E). However, all PSCs containing the GT vector efficiently differentiated into a variety of lineages, suggesting that the presence of the genetic modification did not impinge on differentiation potential.

In conclusion, the GT vector system is a reliable option for the constitutive expression of genes in PSCs and their differentiated derivatives. The vectors complement existing expression systems and therefore will enable the implementation of complex multifaceted genetic modifications that require several independent elements. The ease with which hPSCs can be modified using GT vectors should facilitate tagging, tracking, and overexpression studies in both in vivo and in vitro experiments.

## Experimental Procedures

### Vectors, TALENs, and CRISPRs

Vectors and cell lines described in this report can be obtained by contacting the authors (ed.stanley@mcri.edu.au or andrew.elefanty@mcri.edu.au). Some vectors will also be available through Addgene (see Table S1), and may be located using the search term GAPTrap. The base GT vectors were constructed as follows. Genomic homology arms were amplified from human genomic DNA using primers and cloned into Topoblunt cloning vectors (Invitrogen). Cloned fragments representing the 5′ arm were sequenced to ensure the GAPDH coding sequence was free from PCR-induced mutations. The 5′ arm was cloned upstream of the 3′ arm to create base vectors into which cassettes constituting an IRES (Wu et al., 1994) and selectable marker gene were inserted. Annotated sequences of these base vectors are available in GenBank (GAPTrap-IRESMygro, GAPTrap-IRESMuro, GAPTrap-IRESMeo). The *Mygro*, *Meo*, and *Muro* coding sequences were synthesized to order by GenScript (http://www.genscript.com). Individual variations derived from the base vectors, as described in Table S1, are available upon request from the authors. Reporter genes were introduced into specific base vectors using either conventional cloning or infusion cloning (http://www.clontech.com). In either case, the sequence of the inserted reporter was determined by Sanger sequencing (performed by the Australian Genome Research Facility) and continuity of the translational reading frame from the GAPDH coding region, through the 2A sequence to the inserted reporter, was examined using DNAStar (http://www.dnastar.com) or Snapgene (http://www.snapgene.com) DNA analysis software.

TALENs against the GAPDH locus were designed and assembled by Cellectis Biosearch (http://www.cellectis.com/en/). The TALENs targeted sequences immediately 3′ of the GAPDH stop codon as shown below. The coding sequence of GAPDH is underlined. Sequences bound by the left (5′) and right (3′) TAL effector DNA binding domains are in shown in **UPPER CASE**. Sequences cut by the TALEN pair are *italicized lower case*.

5′…tcttttcatcttctaggtatgacaacgaatttggctacagcaacagggtggtggacctcatggcccacatggcc**TCCAAGGAGTAAGACCC***ctggaccaccagccccag****c****aa***GAGCACAAGAGGAAGAGA**gagaccctcactgctggggagtccctgccacactcagtcccccaccacactgaatctcccctcctcacagtttccatgtagaccccttgaagaggggagg…3′.

Analysis of the TALEN binding sites indicate that this pair of TALENs would also cut two GAPDH pseudogenes located on chromosome 13 and on the X chromosome. PCR analysis failed to detect any transgene integration at these sites, perhaps because antibiotic resistance of transfected cells requires trapping of an active transcription unit. Integration of the GT vectors at these sites may also be inhibited by the imperfect alignment of the pseudogenes with the homology arms of the GAPTrap vectors.

For the CRISPR/CAS9 experiments, synthetic oligonucleotides containing the desired GAPDH protospacer sequence (CTTCCTCTTGTGCTCTTGCT) were annealed to generate a duplex with overhangs compatible with those generated by BbsI digestion of the sgRNA expression plasmid described previously (Howden et al., 2015). To enable in vitro transcription of mRNA encoding CAS9, we inserted an XbaI-NsiI fragment from plasmid hCas9 (Addgene plasmid #41815) containing the entire SpCas9 sequence into the NheI and SbfI sites of the pDNR-Dual vector (Clontech) to generate pDNR-SpCas9.

#### In Vitro Transcription

Capped and polyadenylated in vitro transcribed mRNA encoding CAS9 was generated using the mMESSAGE mMACHINE T7 ULTRA transcription kit (Thermo Fisher) according to the manufacturer's recommendations. Plasmid template was linearized with *PmeI* prior to transcription. LiCl was used to precipitate mRNA before resuspension.

#### Transfection

For targeting experiments using the CRISPR/Cas9 system, transfections were performed using the Neon Transfection System (Thermo Fisher). H9 cells were harvested with TrypLE (Thermo Fisher) 2 days after passaging and resuspended in buffer R at a final concentration of 1 × 10^7^ cells/mL. One hundred microliters of the cell suspension was added to a tube containing circular GT-BFP-IRESMuro vector, mRNA encoding Cas9, and the GAPDH-specific gRNA expression vector. Electroporation was performed in a 100-μL tip using the following conditions: 1,100 V, 30 ms, 1 pulse. Following electroporation cells were transferred to Matrigel-coated plates containing E8 medium supplemented with 5 μM Y-27632 (Tocris), which was omitted in subsequent media changes. Puromycin was added to medium after 4 days to isolate correctly targeted clones.

### Human PSC Culture and Genetic Modification

RM3.5 iPSCs were derived from human foreskin fibroblasts purchased from ATCC and reprogrammed using the hSTEMCCA-loxP four-factor lentiviral vector as described previously (Somers et al., 2010). Following isolation of iPSCs, integrated vector sequences were removed using cre recombinase as described by Davis et al. (2008). PSCs, H9 (Thomson et al., 1998), NKX2-5^GFP/w^ HES3 (Elliott et al., 2011), and the iPSC line, RM3.5, were cultured as described by Costa et al. (2008) and genetic modifications carried out as outlined by Costa et al. (2007) with the following modifications. Cells were electroporated with 10–20 μg of targeting vector and 5 μg of each *GAPDH*-specific TALEN plasmid. GAPTrap vectors were linearized with *Pac1* prior to electroporation, or delivered as uncut circles. Antibiotic selection was commenced between 2 and 5 days post electroporation, using either 75 μg/mL hygromycin (GT-Myrgo vectors), 75 μg/mL G418 (GT-Meo vectors), or 1 μg/mL puromycin (GT-Muro vectors). G418 and hygromycin selection were continued for 7–14 days while puromycin selection was applied for 2–7 days. Antibiotic-resistant clones were picked, expanded, and cryopreserved (Costa et al., 2007). For vectors without a selectable marker, hPSCs were electroporated, and GFP-expressing single cells were isolated by FACS after 2–5 days using a BD Influx cell sorter (http://www.bdbiosciences.com/in/instruments/influx/index.jsp) as described by Davis et al. (2008). Individual GFP-expressing clones were expanded for further analysis.

### Analysis of Genomic Integrity, Characterization, and Differentiation

Genomic integrity of selected genetically modified lines was assessed using the Illumina HumanCytoSNP-12 v2.1 array and was performed by the Victorian Clinical Genetics Service, Royal Children's Hospital (Melbourne) or by standard karyotype analysis as described previously. Selected individual clones were examined for expression of EPCAM and CD9 expression using flow cytometry (Figure S1A; antibodies are listed in Table S2). GT-lacZ iPSC lines were tested for pluripotency using a teratoma assay (Gropp et al., 2012). Endoderm differentiation was performed in AEL medium (Ng et al., 2008) using the factor combinations and timing reported by Loh et al. (2014). Hematopoietic differentiation was performed using the Spin EB method (Ng et al., 2008). Cardiomyocytes were generated using a modification of the method reported by Lian et al. (2012) and Skelton et al. (2014), with the additional change that 5 μM IWR1 (Sigma i0161) was used in place of IWP2. TH-positive neurons were generated as previously described (Kriks et al., 2011) except that cells were cultured on mTESR2 and differentiation initiated on laminin-521 at 10 μg/ml (Biolamina, as per manufacturer’s instructions) or vitronectin at 10 μg/ml (STEMCELL Technologies).

### Flow Cytometry and Western Blotting

For flow cytometry analysis of cell surface marker expression, embryoid bodies or adherent cultures were washed with PBS and dissociated with TrypLE Select (www.thermofisher.com/order/catalog/product/12563011) for 2–10 min at 37°C to generate a single cell suspension. TrypLE Select digestion was terminated by addition of 5–10 volumes of PBS and the cells collected by centrifugation. The cell pellet was resuspended in FACS buffer (PBS with 2% fetal calf serum) with the diluted antibody (see Table S2) added. Antibody binding was allowed to proceed for up to 30 min on ice. Samples were then washed twice with 5 mL of PBS and the pellet resuspended in FACS buffer containing 1 μg/ml propidium iodide. Samples were analyzed with a Becton Dickinson FACSCalibur or LSRFortessa. Cells for intracellular flow cytometry were collected as above and then processed using a BD Cytofix/Cytoperm Kit according to the manufacturer’s instructions. Cells were labeled with a mouse anti-luciferase antibody (Abcam), which was subsequently detected with an APC-conjugated goat anti-mouse secondary antibody, as described by Mossman et al. (2005).

### Bioluminescent Imaging and Teratoma Assays

For in vivo imaging, approximately 10^6^ GT-Luc2 PSCs were transplanted subcutaneously into NOD/SCID/IL2Rγ-null mice (Shultz et al., 2005). Eight days post transplantation, mice were administered 150 μg/g of VivoGlo luciferin (Promega) by intraperitoneal injection. Five minutes after administration of luciferin, mice were anesthetized by inhalation of isoflurane (2% in oxygen) for 5 min and then imaged using a Xenogen IVIS-200 imaging system (Caliper). Images obtained were processed using Living Image software (Caliper) to normalize luciferase expression data collected across multiple time points.

Teratomas for GT-lacZ iPSCs were initiated by subcutaneous injection of 10^6^ cells as described by Gropp et al. (2012). After 4–6 weeks, mice harboring a palpable mass at the injection site were killed by cervical dislocation, and the teratoma resected and fixed in 4% paraformaldehyde (PFA). Teratoma fragments were stained with X-gal as described, post-fixed in 4% PFA, embedded in paraffin, and processed for histology. Sections were stained with eosin. Mouse work was covered by institutional animal ethics approvals A788 (Murdoch Children’s Research Institute) and SOBSA/MIS/2009/107 (Monash University).

### Immunofluorescence and Luciferase Assays

Immunofluorescence for the neural marker genes TH and FOXA2 was performed as described by Niclis et al. (2013) using the antibody dilutions described in Table S2. Assays for *Gaussia* luciferase was conducted using coelenterazine (CTZ) substrate (Nanolight) as described by the supplier. Approximately 10^3^ cells representing three independently derived GT-GLuc iPSCs (and controls) were assayed without lysis and light emission measured at 475 nm.

## Author Contributions

T.K., T.L., E.Q., A.M., M.C., K.S., J.V.C., S.E.H., A.G., and D.A. generated vectors and/or cell lines. T.K., J.C., R.C., E.Q., F.F.B., E.N., D.A., P.F., Z.L., and J.C.N. performed differentiation experiments and/or characterized differentiated cells. T.K. and S.R.L. performed western blotting experiments. M.H.L., A.R., D.A.E., A.J.P., L.H.T., C.L.P., M.C., P.F., and S.R.L. provided supervision and/or critical assessment of the final manuscript. A.G.E. and E.G.S. designed and supervised implementation of the study and assembly and critique of the final manuscript.

## Supplemental Information

Document S1. Figures S1 and S2 and Tables S1 and S2 Document S1. Figures S1 and S2 and Tables S1 and S2

Document S2. Article plus Supplemental Information Document S2. Article plus Supplemental Information
