# Supplementary material for: GAPTrap: A Simple Expression System for Pluripotent Stem Cells and Their Derivatives
Source: Stem Cell Reports. 2016 Sep 1;7(3):518–26. doi: 10.1016/j.stemcr.2016.07.015 (PMC5032031; doi:10.1016/j.stemcr.2016.07.015)
Supplement: Document S1. Figures S1 and S2 and Tables S1 and S2 [file mmc1.pdf]

Stem Cell Reports, Volume 7

Supplemental Information

GAPTrap: A Simple Expression System for Pluripotent Stem Cells and Their Derivatives

Tim Kao, Tanya Labonne, Jonathan C. Niclis, Ritu Chaurasia, Zerina Lokmic, Elizabeth Qian, Freya F. Bruveris, Sara E. Howden, Ali Motazedian, Jacqueline V. Schiesser, Magdaline Costa, Koula Sourris, Elizabeth Ng, David Anderson, Antonietta Giudice, Peter Farlie, Michael Cheung, Shireen R. Lamande, Anthony J. Penington, Clare L. Parish, Lachlan H. Thomson, Arash Rafii, David A. Elliott, Andrew G. Elefanty, and Edouard G. Stanley

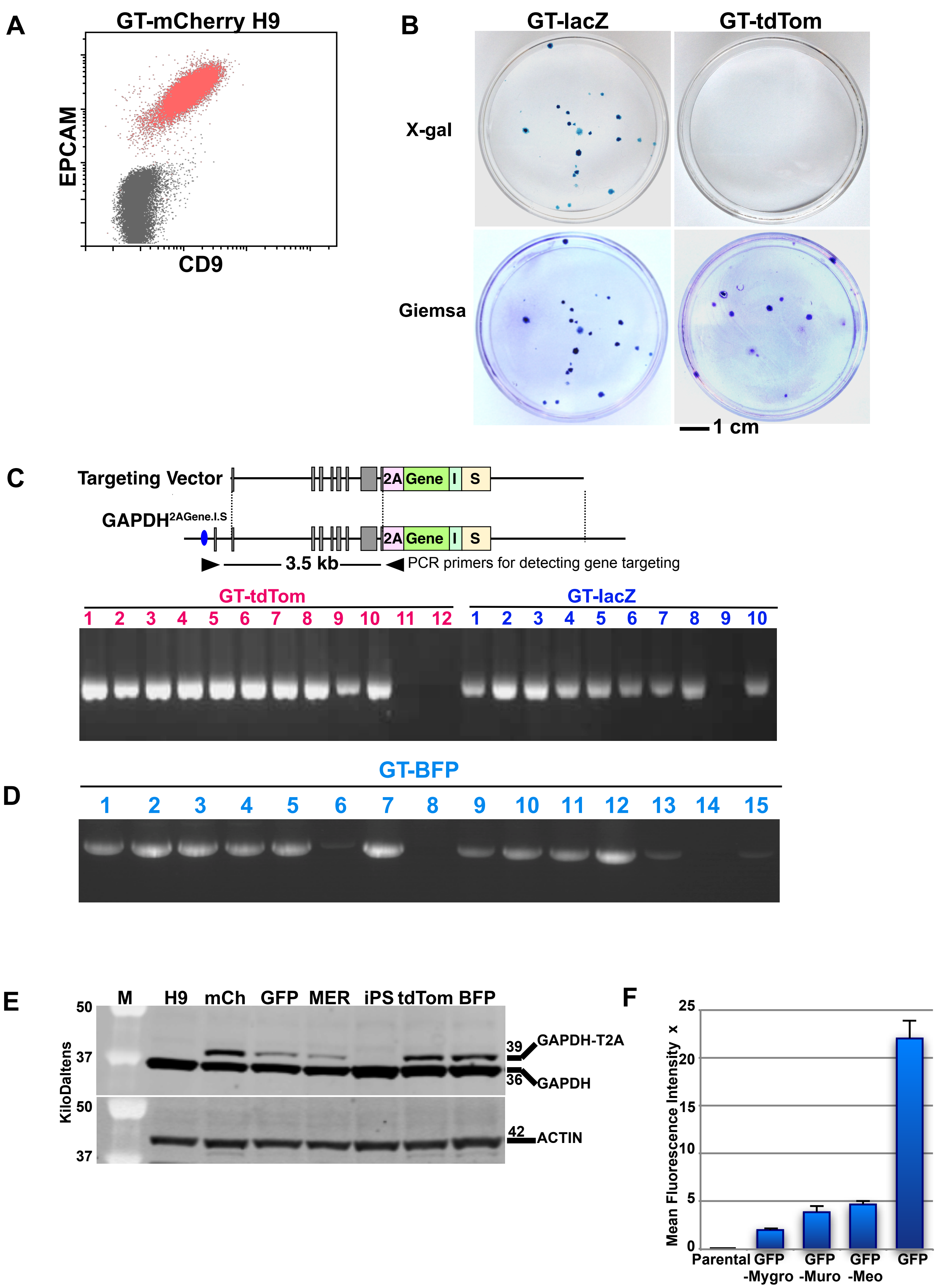
A
GT-mCherry H9
EPCAM
CD9
B
GT-lacZ
GT-tdTom
X-gal
Giemsa
1 cm
C
Targeting Vector
2A
Gene
I
S
GAPDH2AGene.I.S
3.5 kb
PCR primers for detecting gene targeting
GT-tdTom
1 2 3 4 5 6 7 8 9 10 11 12
GT-lacZ
1 2 3 4 5 6 7 8 9 10
D
GT-BFP
1 2 3 4 5 6 7 8 9 10 11 12 13 14 15
E
M H9 mCh GFP MER iPS tdTom BFP
50
37
KiloDaltens
50
37
39
GAPDH-T2A
36
GAPDH
42
ACTIN
F
Mean Fluorescence Intensity x
0
5
10
15
20
25
Parental
GFP -Mygro
GFP -Muro
GFP -Meo
GFP

Figure S1. A. Flow cytometry analysis of GT-mCherry H9 hESCs labelled with anti-CD9 and anti-EPCAM antibodies (red) or with isotype control antibodies (Grey), showing maintenance of stem cell marker expression following genetic manipulation. **B.** Tissue culture plates containing puromycin resistant colonies of GT-lacZ or GT-TdTom iPSCs stained for beta-galactosidase (upper panels) to reveal cells expressing the GT transgene and then re-stained with Giemsa to reveal all colonies. Note that transgene expression is usually indicative of correct gene targeting. **C.** PCR analysis of individual puromycin resistant colonies harbouring either GT-TdTom of GT-lacZ transgenes using the strategy indicated (upper line). These results indicate 19 out of 22 clones analysed were correctly targeted. **D**. PCR analysis of GT-BFP clones generated with CRISPR assisted gene targeting using the screening strategy indicated in C. This analysis shows at least 12 of 15 clones are correctly targeted. **E.** Western blot analysis of GAPDH expression in wild type or GapTrap transgenic PSC lines showing reduced levels of GAPDH-T2A protein relative to GAPDH produced from unmodified alleles. The lower panel is derived from the same western blot in the upper panel but probed with an anti-beta actin antibody. The sizes of molecular standards in kilodaltons are shown on the left hand side whilst sizes and positions of GAPDH, GAPDH-T2A (from the modified allele) and beta Actin are indicated on the right. From left to right, Molecular weight markers (M), H9 hESCs (H9), GT-mCherry-Mygro H9 hESCs (mCh), GT-GFP-Neo H9 hESCs (GFP), GT-MYC:ER H9 hESCs (MER), RM3-5 iPSCs, GT-Tandom Tomato RM3-5 iPSCs (TdTom), GT-mTagBFP2 (BFP). **F.** GFP expression from different version of the GT vectors containing IRES-selectable marker cassettes as indicated. Note that right-most column represents GT-GFP cells containing no IRES-selectable marker gene. Error bars represent the standard error of the mean derived from the analysis of greater than 3 independently derived cell lines.

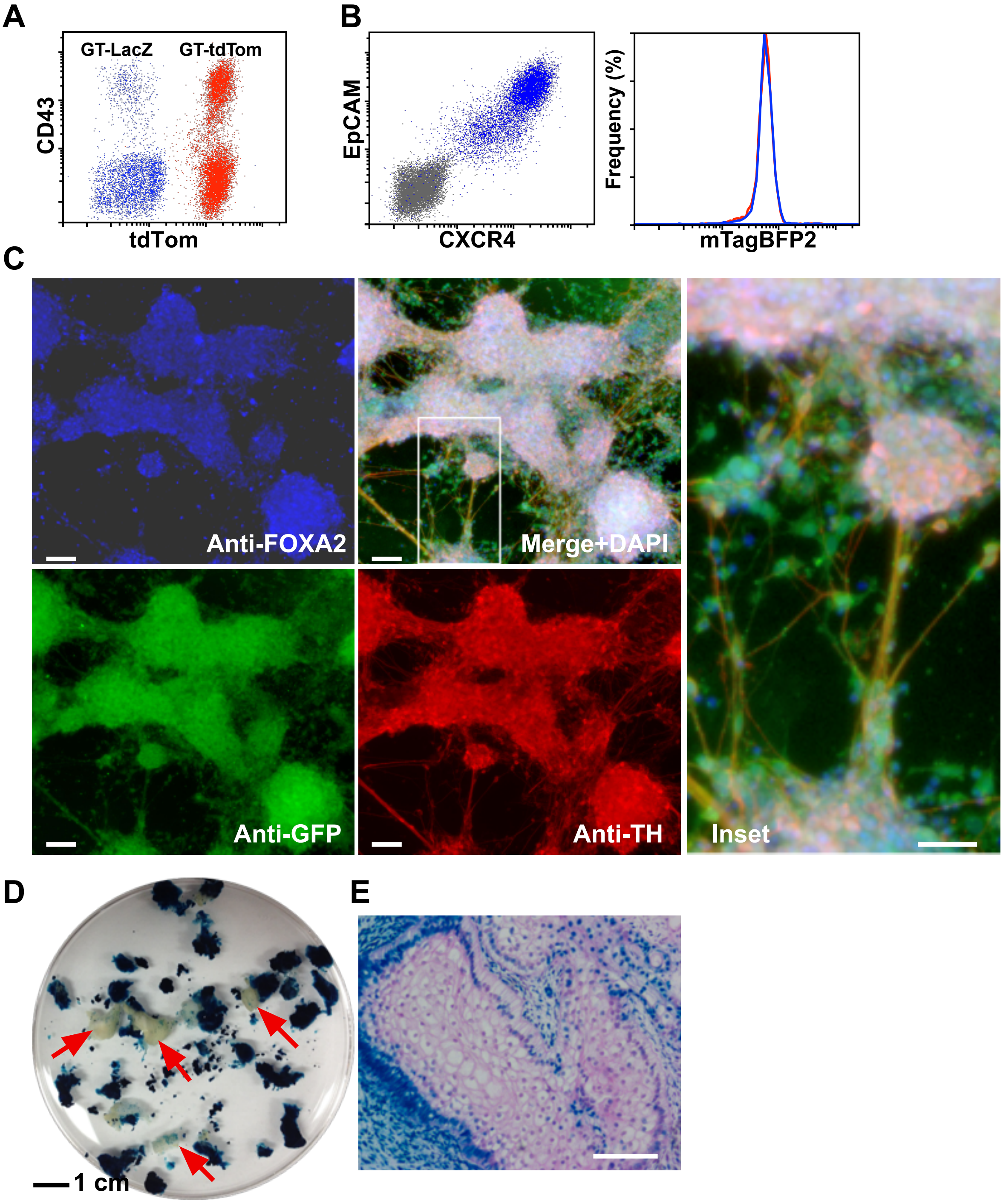

A
GT-LacZ
GT-tdTom
CD43
tdTom
B
EpCAM
CXCR4
Frequency (%)
mTagBFP2
C
Anti-FOXA2
Merge+DAPI
Anti-GFP
Anti-TH
Inset
D
1 cm
E

Figure S2. **A**. Flow cytometry analysis showing robust expression of TdTom in CD43+ blood cells differentiated from GT-TdTom iPSCs. **B.** Left panel: differentiation day 5 CXCR4+EpCAM+ definitive endoderm cells (blue) and cells stained with isotope control antibodies (grey) derived from GT-mTagBFP2 iPSCs. Right panel: Histogram plot showing consistent BFP expression in the endoderm populations derived from 2 independent GT-BFP clones. **C.** Immunofluorescence images showing uniform and ubiquitous expression of GFP (detected with and anti-GFP antibody) in midbrain dopamine neurons, identified by FOXA2 and Tyrosine Hydroxylase (TH) immunofluorescence, derived from GT-GFP iPSCs. Cell nuclei were identified by DAPI staining. Scale bars = 100 µm. **D**.Teratoma fragments derived from GT-lacZ iPSCs following fixation and staining with X-gal. Examples of mouse connective tissue are indicated by red arrows. All teratoma fragments displayed uniform intense surface staining but histological sectioning (**E**) revealed that X-gal did not penetrate into the deeper interior structures of teratomas. Scale bars = 100 µm.

Supplementary Table 1						
**Vector**	**Addgene**	**PSC line**	**Reporter**	**Selection**	**MFI***	**Karyotype#**
**GT-GFP-INeo**	**C.A**	H9	eGFP	G418	N.D	Normal
**GT-GFP-IMeo**	82502	RM3.5	eGFP	G418	4690	Normal
**GT-GFP-IMuro**	82504	RM3.5	eGFP	Puromycin	3879	Not tested
**GT-GFP-IMygro**	82503	RM3.5	eGFP	Hygromycin B	1985	Not tested
**GT-GFP**	82506	RM3.5	eGFP	None	22061	Not tested
**GT-mCherry-IMygro**	82505	H9/RM3.5	mCherry	Hygromycin B	13922	Normal/Not tested
**GT-TdTom-IMuro**	82355	RM3.5	Tandom Tomato	Puromycin	24540	Not tested
**GT-mTagBFP2-IMuro**	82335	RM3.5	mTagBFP2	Puromycin	8974	Normal
**GT-LacZ-IMuro**	82507	RM3.5	LacZ	Puromycin	N/A	Normal
**GT-Gluc-IMuro**	**C.A**	RM3.5	Gaussia Luciferase	Puromycin	N/A	Not tested
**GT-Luc2-IMeo**	82509	H9	Firefly Luciferase	G418	N/A	Normal
**GT-Clover-T2A-Muro**	82334	RM3.5	Clover	Puromycin	7150	Not tested

I, IRES. *Relative Average Mean fluorescence intensity. The MFI of unmodified hPSCs was less than 300. N/A, not applicable, N.D., not determined. C.A., contact authors for further information. # Indicates at least one cell line was derived that had a normal karyotype. Accession numbers for vectors submitted to Addgene are indicated.

**Supplementary Table 2**				
**Antibody**	**Supplier, Cat#**	**Technique**	**Figure**	**Dilution**
EpCAM- APC	Biolegend, 324208	FC	2D, S1A	1:30
CD9-FITC	Becton Dickonson, 555371	FC	S1A	1:20
CXCR4-BV	Biolegend, 306518	FC	2D	1:30
CD45-PE	Becton Dickonson, 555483	FC	2B	1:50
FOXA2	SantaCruz, Sc-6554	IF	S2B	1:200
GFP	Abcam, Ab13970	IF	S2B	1:1500
TYROSINE HYDROXYLASE	Pel-Freeze, P40101-0	IF	2F, S2C	1:400
Secondary 647 for FOXA2	Jackson ImmunoResearch, 705-605-147	IF	S2C	1:400
Secondary 488 for GFP	Jackson ImmunoResearch, 703-545-155	IF	S2C	1:400
Secondary 488 for TH	Jackson ImmunoResearch, 711-545-152	IF	2F	1:400
Secondary 550 for TH	Abcam, Dylight AB96892	IF	S2C	1:400
luciferase (*luc2*)	Abcam, ab16466	IFC	1D	1:400
Goat-anti-Mouse-APC	Becton Dickonson, 550826	IFC	1D	1:200
*G*luciferase (*Gluc)*	Prolume Ltd, 401M	FC	1E	1:25
Goat-anti-Mouse-APC	Biolegend Poly4053, 405308	FC	1E	1:50
CD43-APC	BioLegend, 343206	FC	S2A	1:30
Beta-Actin	Novus, NB600-501	WB	S1	1:5000
GAPDH	Cell Signaling, 5174	WB	S1	1:5000

FC, flow cytometry. IFC, intracellular flow cytometry. WB, western blot. IF, immunofluorescence.
